# Glucagon-like peptide-1 (GLP-1) receptor agonists for headache and pain disorders: a systematic review

**DOI:** 10.1186/s10194-024-01821-3

**Published:** 2024-07-12

**Authors:** Wael Halloum, Yousef Al Dughem, Dagmar Beier, Lanfranco Pellesi

**Affiliations:** 1 Clinical Pharmacology, Pharmacy and Environmental Medicine, Department of Public Health, University of Southern Denmark, Campusvej 55, Odense, 5230 Denmark; 2 Department of Neurology, Odense University Hospital, Odense, Denmark; 3 Department of Clinical Research, University of Southern Denmark, Odense, Denmark

**Keywords:** Diabetic neuropathy, Irritable bowel syndrome, Migraine, Neuropathic pain, Osteoarthritis

## Abstract

**Background:**

Glucagon-like peptide-1 (GLP-1) plays a crucial role in metabolic disorders by enhancing insulin secretion, inhibiting glucagon release, and slowing gastric emptying, thereby improving glycemic control. In recent years, GLP-1 role in neuronal pathways has expanded its therapeutic potential. We aim to comprehensively evaluate the relevance of GLP-1 in headache and pain disorders.

**Methods:**

A systematic literature search was conducted on PubMed and Embase (Ovid) databases using the search terms “GLP-1” and “pain”. Animal and human studies published in English language were included. Abstracts, reviews, and articles on other disorders than “pain” were excluded.

**Results:**

The search strategy identified 833 hits, of which 42 studies were included in the final review. The studies were categorized into four groups: inflammatory pain and osteoarthritis, headaches, neuropathic pain and diabetic neuropathy, and visceral pain and irritable bowel syndrome. GLP-1 receptor (GLP-1R) agonists, like liraglutide, have shown analgesic effects by modulating pain hypersensitivity in animal models of inflammatory and neuropathic pain. GLP-1 is involved in migraine mechanisms and GLP-1R agonists are beneficial in individuals with idiopathic intracranial hypertension. Additionally, GLP-1R agonists reduce visceral hypersensitivity and ameliorate symptoms in patients with irritable bowel syndrome.

**Conclusions:**

The therapeutic scope of GLP-1R agonists is expanding beyond traditional metabolic targets, highlighting its potential for headache and pain disorders. Engineering bimodal molecules that integrate GLP-1R agonism with specific pain-related mechanisms may offer innovative therapeutic options.

## Background

Glucagon-like peptide-1 (GLP-1) is a peptide hormone known for its role in regulating glucose homeostasis and satiety. Synthesized in the intestinal L-cells, GLP-1 is secreted in response to food intake, stimulating insulin secretion and inhibiting glucagon release [1]. GLP-1 enhances glucose-dependent insulin secretion from pancreatic beta cells, inhibits glucagon release from alpha cells, and slows gastric emptying, thereby aiding in blood glucose regulation. The GLP-1 receptor (GLP-1R) is a G-protein-coupled receptor widely expressed in various tissues, including the pancreas, brain, and gastrointestinal tract. Upon binding to GLP-1, GLP-1R activates intracellular signaling pathways, such as cyclic adenosine monophosphate (cAMP) and protein kinase A (PKA), which mediate its physiological effects. Due to its potent effects on glucose homeostasis and appetite regulation, GLP-1R agonists are treatments for type 2 diabetes and obesity [2, 3]. In addition, GLP-1 has significant effects on the nervous system [4, 5]. GLP-1R is expressed in various brain regions, including the hypothalamus, cortex, and hippocampus, as well as peripheral nervous tissues [6]. The widespread distribution implicates GLP-1 in several neural processes, including neuroprotection, synaptic plasticity, and modulation of neuroinflammation [7]. These properties have sparked interest in GLP-1R agonists as potential treatments for neurological disorders, such as Parkinson’s disease (PD) and Alzheimer’s disease (AD). PD leads to debilitating motor and non-motor symptoms and is characterized by the progressive degeneration of dopaminergic neurons in the substantia nigra. Neuroinflammation and oxidative stress are key pathological features of PD [8, 9]. Pre-clinical studies have demonstrated anti-PD effects of GLP-1R agonists and clinical trials are underway to evaluate the efficacy of GLP-1R agonists in slowing disease progression and alleviating symptoms in PD patients [10, 11]. Similarly, GLP-1R agonists are candidate therapies for AD due to their role in modulating neuroinflammation and β-amyloid accumulation. Experimental models have shown that GLP-1R activation can improve cognitive function and reduce amyloid plaque burden, suggesting potential disease-modifying effects [12, 13]. GLP-1R agonists may influence other neurological pathways, particularly in pain conditions. This systematic review aims to comprehensively evaluate the role of GLP-1 in headache and pain disorders. By emphasizing the expanding therapeutic scope of GLP-1 beyond traditional metabolic targets, we elucidate the current evidence and potential mechanisms of GLP-1R agonists in neurological conditions other than PD and AD.

## Methods

We performed a systematic literature search identifying articles reporting original data on GLP-1 and pain, including headaches. We conducted the literature search on PubMed and Embase (Ovid) in December 2023 and updated it in June 2024. We used the following search terms: “GLP-1” and “pain”. Both animal and human studies published in English were included. Original studies that did not investigate pain as a primary and/or secondary outcome were excluded. Abstracts, reviews, editorials, and other articles without original data were also excluded. Additionally, we considered articles from the reference lists of relevant studies and literature known to be pertinent by the authors.

### Data extraction

Titles and abstracts of retrieved records were screened independently and separately for obvious exclusions. Exclusion of records was performed using voting based on a majoritarian system. Data extraction was conducted independently by LP and WH/YAD, who reviewed full texts of selected articles to extract relevant data on study design, population, interventions, outcomes, and results. Discrepancies were resolved through collegial discussions. We used Endnote (Endnote 21) software to manage references, identify duplicate records, and remove them. We followed PRISMA guidelines for systematic reviews to ensure transparency and completeness in our reporting [14].

## Results

**Fig. 1:**
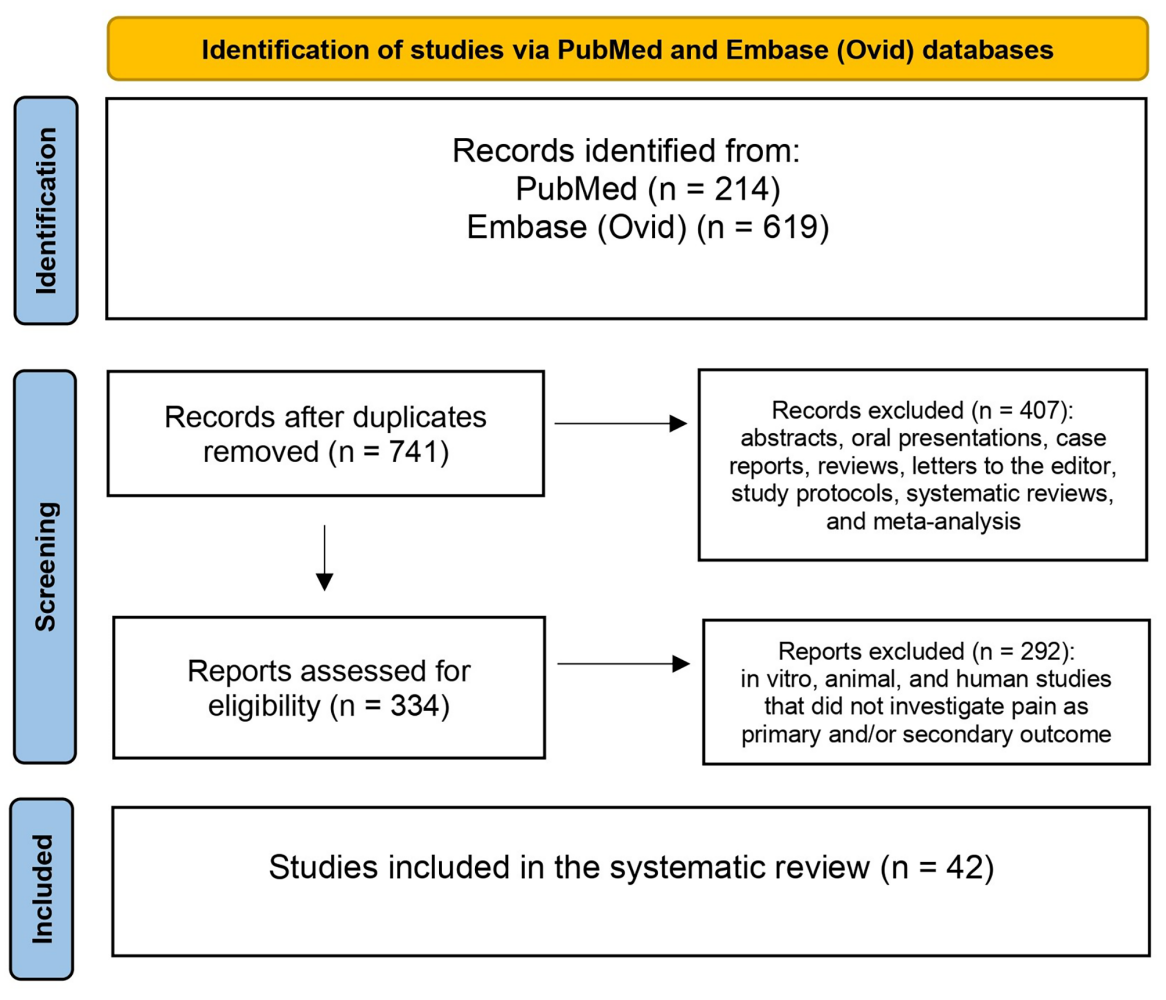
Flowchart of the systematic review

**Fig. 2:**
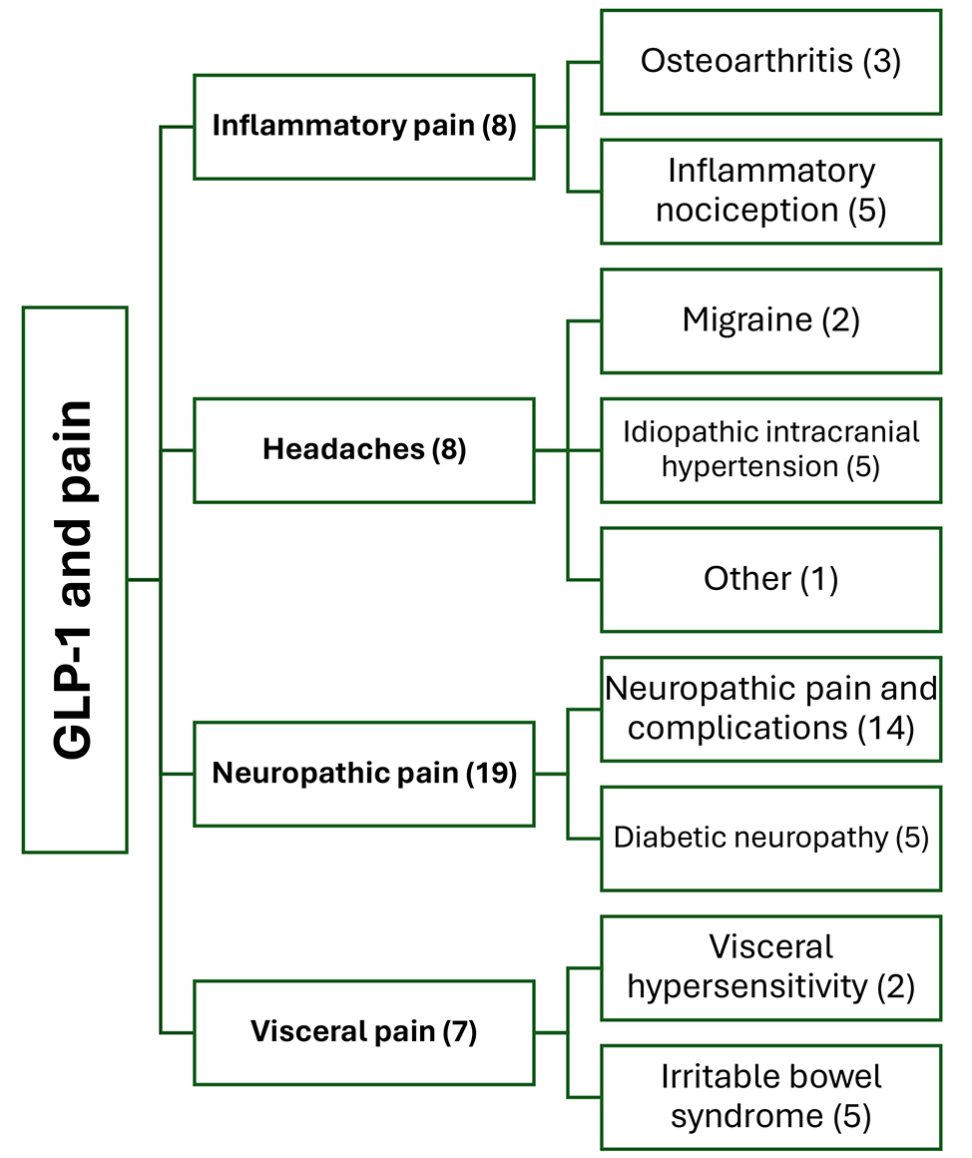
Overview and categorization of the studies evaluating the role of glucagon-like peptide 1 (GLP-1) in headache and pain disorders

Our search strategy identified 833 hits of which 42 studies were included in the final review. After excluding 92 hits as duplicates, another 282 hits were excluded because they were reviews, abstracts, oral presentations and letters to the editors. We also excluded case reports (*n* = 97), study protocols, systematic reviews and meta-analysis (*n* = 28). After full-text assessment, we excluded studies on diabetes mellitus (*n* = 154), studies on Parkinson or neurodegenerative conditions (*n* = 8), and in vitro, animal and human studies that did not investigate pain as primary and/or secondary outcome (*n* = 130). In total, 42 animal and human studies were included in the final review (Fig. 1). The identified studies were further divided in four categories: (1) inflammatory pain and osteoarthritis, (2) headaches, (3) neuropathic pain and diabetic neuropathy, and (4) visceral pain and irritable bowel syndrome (IBS) (Fig. 2). Experimental compounds used to test GLP-1 role in headache and pain disorders are listed in Table 1. Human and animal experimental techniques are described in Table 2.

**Table 1:** Experimental compounds used to test glucagon-like peptide 1 (GLP-1) role in headache and pain disorders

GLP-1 and GLP-1 analogs	Exendin 4, GLP-1, GLP-1 (7–36 amide) and ROSE-010
**GLP-1 receptor agonists**	Exenatide, liraglutide, semaglutide and WB4-24
**Dipeptidyl peptidase 4 (DPP-4) inhibitors**	Evogliptin tartrate, diprotin A, PKF275-055, teneligliptin and vildagliptin
**Natural products with a GLP-1 receptor agonistic activity**	Morroniside, geniposide, *Lamiophlomis rotata* and its principle effective iridoid glycoside (shanzhiside methylester)

**Table 2:** Human and animal experimental techniques used to study the role of glucagon-like peptide (GLP-1) in headache and pain disorders

	Animal studies	Human studies
**Inflammatory pain**	Mouse model of sodium monoiodoacetate osteoarthritis	Randomized, placebo-controlled trial
	Carrageenan-induced, formalin-induced and Complete Freund´s Adjuvant (CFA)-induced peripheral inflammation	Prospective observational study
**Headaches**	Hydrocephalus rat model with raised intracranial pressure	Randomized, placebo-controlled trial
	Nitroglycerin-induced model of migraine	Case-control study
**Neuropathic pain**	Formalin tests	
	Bone cancer pain model	
	Spared nerve injury, partial sciatic nerve transection and spinal nerve ligation models	
	Nicotinamide- and streptozotocin-induced model of diabetic pain	
**Visceral pain**	Model of visceral hypersensitivity induced by lipopolysaccharide, repeated water avoidance stress and intra-colonic infusion of acetic acid	Randomized, placebo-controlled trial
	Model of irritable bowel syndrome	

### Inflammatory pain and osteoarthritis

We identified eight studies exploring the interplay between GLP-1 and inflammatory pain [15–22]. Liraglutide, a GLP-1 analog, emerges as a promising therapeutic agent in this context. In a murine model of osteoarthritis, intra-articular injection of liraglutide mitigated pain-associated behaviors induced by sodium monoiodoacetate [15]. In vitro investigations revealed its capacity to downregulate inflammatory gene expression and suppress the synthesis of interleukin 6 (IL-6), prostaglandin E2 and nitric oxide in chondrocytes and macrophages in a dose-dependent manner. Moreover, liraglutide elicited a phenotypic shift in macrophages from proinflammatory M1 to anti-inflammatory M2, while also inhibiting the expression of enzymes implicated in cartilage degradation. Another study in rats showcased the efficacy of liraglutide in alleviating acute peripheral inflammation induced by carrageenan, either alone or in combination with tramadol [16]. Mechanistic insights revealed reductions in swelling and paw temperature, alongside elevations in anti-inflammatory interleukin 10 (IL-10) and total antioxidant status within inflamed tissues. WB4-24, a non-peptide GLP-1 receptor agonist, demonstrated dose-dependent attenuation in mouse models of acute and chronic inflammatory nociception via the release of β-endorphins from spinal microglia [17]. Further investigations involving dipeptidyl peptidase 4 (DPP-4) inhibitors such as evogliptin tartrate, diprotin A, and vildagliptin underscored the analgesic properties of GLP-1 analogs through modulation of the endogenous opioid system [18–20]. Human studies exploring the analgesic efficacy of liraglutide in knee osteoarthritis yielded mixed results, suggesting that clinical benefits may derive from weight loss rather than direct analgesic mechanisms [21, 22]. A randomized, placebo-controlled trial was conducted in people with knee osteoarthritis and a Body Mass Index (BMI) > 27 [21]. A total of 156 trial participants were randomized to receive daily liraglutide (30 mg) (*n* = 80) or placebo (*n* = 76) for 52 weeks. Pain was measured using the Knee Injury and Osteoarthritis Outcome Score measurement scale. Compared to placebo, patients treated with liraglutide lost weight but did not experience less knee pain. A prospective, observational, multicenter study was conducted in more than 40,000 Chinese adults with knee osteoarthritis and type 2 diabetes mellitus [22]. Patients taking GLP-1R agonists lost weight and had a lower risk of knee surgery than patients not taking GLP-1R agonists. The use of GLP-1R agonists was associated with fewer intra-articular injections of steroids and a lower cartilage loss velocity from the medial femoral joint, but the direct effects of GLP-1R agonist, apart from the weight loss-mediated pathway, did not reach statistical significance.

### Headaches

Seven studies were identified concerning headache, migraine, and idiopathic intracranial hypertension (IIH) [23–29]. In rats, GLP-1R expression is found in microglial cells within the trigeminocervical complex [23]. In a rat migraine model, liraglutide mitigated nitroglycerin-induced sensitization of the trigeminocervical complex, leading to reduced expression of pain mediators such as calcitonin gene-related peptide and c-Fos [23]. Moreover, liraglutide suppressed the production of pro-inflammatory molecules interleukin 1β (IL-1β) and tumor necrosis factor alpha (TNF-α) via the phosphoinositide 3kinase/serine-threonine kinase (PI3K/Akt) signaling pathway. A second study in rats highlighted liraglutide´s role in promoting the release of IL-10, an anti-inflammatory cytokine that was effective in relieving migraine-associated pain [24]. GLP-1R is present in the human choroid plexus, where GLP-1R analogs reduces cerebrospinal fluid secretion and intracranial pressure by increasing the intracellular concentration of cyclic adenosine monophosphate and inhibiting the Na^+^/K^+^ ATPase pump in the choroid plexus [25]. GLP-1R agonists have been tested for their ability to promote weight loss and reduce headache in patients with IIH. A single-center, case-control pilot study included 39 participants with a BMI ≥ 30 kg/m² [26]. Participants (*n* = 13) were treated with semaglutide or liraglutide in addition to standard body weight management, while a control group (*n* = 26) received standard body weight management. The group treated with GLP-1R agonists achieved significantly greater weight loss after six months compared to the control group (-12.0% vs. -2.8%). Furthermore, the group treated with GLP-1-R agonists experienced fewer headache days than the other group while the dose of acetazolamide was reduced. A double-blind, placebo-controlled trial allocated women with active IIH to 12-week dosing of either placebo (*n* = 7) or exenatide (*n* = 7), a GLP-1R agonist [27]. The primary outcome was the difference in intracranial pressure between exenatide and placebo at 2.5 h, 24 h, and 12 weeks. A meaningful reduction in intracranial pressure has been reported at all time points in the exenatide group as compared to the placebo group. Mean monthly headache days reduced significantly in the exenatide arm (− 7.7 days) compared to the placebo arm (-1.5 days), with no significant difference between exenatide and placebo groups at 12 weeks. There was no significant change in BMI within the exenatide group at 12 weeks, indicating that the effect on intracranial pressure was not driven by reduction in body weight, but likely by direct effect of exenatide to modulate intracranial pressure at the choroid plexus. A placebo-controlled exploratory study showed that exenatide treatment for 12 weeks did not affect cognitive function in women with IIH [28]. The abrupt cessation of GLP-1R agonists in individuals with metabolic disorders may lead to adverse effects, as seen in a case where a patient developed IIH after discontinuing duraglutide [29]. A study in healthy volunteers explored the vasodilatory and headache-inducing properties of GLP-1, revealing no significant differences in post-infusion headache between GLP-1 and placebo [30].

### Neuropathic pain and diabetic neuropathy

We identified 19 animal studies evaluating the role of GLP-1 in neuropathic pain [31–49]. Intrathecal administration of GLP-1R agonists such as GLP-1(7–36) and exenatide alleviated hypersensitivity in models of formalin-induced, peripheral nerve injury-induced, bone cancer-induced, and diabetes-induced pain in mice and rats [31]. GLP-1(7–36) and exenatide activated GLP-1Rs expressed on microglial cells in the spinal dorsal horn, which were significantly upregulated following peripheral nerve injury. These effects were completely prevented by GLP-1R antagonism and GLP-1R gene knockdown, although acute nociceptive responses were unaffected. Electroacupuncture demonstrated similar findings, relieving pain hypersensitivity in rats with spared nerve injury [32]. In the ipsilateral dorsal horn, electroacupuncture reduced ionized calcium-binding adapter molecule 1 (Iba-1) and glial fibrillary acidic protein (GFAP) levels while increasing the expression levels of GLP-1 and GLP-1R. In a rat spinal nerve ligation model, exenatide exhibited antiallodynic effects on neuropathic pain [33]. Differential gene expression analysis indicated that exenatide could normalize the aberrant expression of 591 genes in the spinal dorsal horn caused by nerve injury, particularly those related to inflammatory signaling via TNF-α and Toll-like receptors. In a similar model, intrathecal injections of exenatide inhibited thermal hyperalgesia and mechanical allodynia by enhancing spinal microglial expression of β-endorphins, IL-10 and interleukin 4 (IL-4) [34, 35]. Teneligliptin (TEN), a DPP-4 inhibitor, demonstrated mild analgesic effects against acute pain and significant effects against neuropathic pain in a rat model induced by partial transection of the sciatic nerve [36]. By preventing the breakdown of GLP-1 and prolonging its circulation, TEN antinociceptive action was partially counteracted by the GLP-1R antagonist exendin-3, suggesting GLP-1R-independent mechanisms. TEN significantly reduced glial fibrillary acidic protein immunoreactivity and astrocyte activation, implying that its analgesic effects are associated with suppression of spinal astrocytes and neuroinflammation [36, 37]. Morroniside, a GLP-1R agonist, reduced mechanical allodynia and thermal hyperalgesia in a dose-dependent manner in a rat model of neuropathic pain, with peak effects within 1 h and lasting over 4 h [38]. Repeated daily injections for seven days did not induce tolerance to its analgesic effects. In a more detailed study, morroniside enhanced IL-10 and β-endorphin gene expression in the spinal lumbar enlargements and cultured microglia of neuropathic rats [39]. Neutralization of spinal IL-10 or β-endorphin, or blockade of the µ-opioid receptor, completely reversed morroniside-induced mechanical antiallodynia. Geniposide, a major iridoid glycoside of Gardenia jasminoides and a GLP-1R agonist, dose-dependently reduced formalin-induced pain in rats without generating antinociceptive tolerance [40]. *Lamiophlomis rotata*, a Tibetan herb containing iridoid glycosides with GLP-1R agonist activity, blocked formalin-induced hyperalgesia, peripheral nerve injury- and bone cancer-induced mechanical allodynia [41]. The herb reduced pain by 50 to 80% at doses between 130 and 250 mg/kg without leading to antiallodynic tolerance. Shanzhiside methyl ester (SM), the major iridoid glycoside of *Lamiophlomis rotata*, demonstrated dose-dependent and long-lasting anti-allodynic effects in neuropathic rats without inducing tolerance [42]. SM significantly induced β-endorphin expression and activated p38 mitogen-activated protein kinase signaling in the spinal dorsal horn and primary microglia. Regarding neuropathic pain-related complications, exenatide attenuated memory deficits induced by neuropathic pain in rats [43, 44]. Neuropathic pain may impair memory by reducing GLP-1R levels in the hippocampus [45]. Immunohistochemical staining and western blot assays revealed increased microglial cells and activated astrocytes in the dentate gyrus of the hippocampus in rats with neuropathic pain, along with elevated expression of TNF-α, IL-1β, and IL-6 [43, 44]. Activation of GLP-1R ameliorated these memory deficits through regulation of adenosine monophosphate-activated protein kinase (AMPK) and nuclear factor-κB (NF-κB) pathway, reducing hippocampal neuroinflammation. Five studies focused specifically on the role of GLP-1 in diabetic neuropathy [46–50]. Liraglutide improved nociceptive thresholds and mitigated histopathological damage of the sciatic nerve in a rat model of diabetic neuropathy induced by nicotinamide and streptozotocin [46]. Liraglutide normalized the content of malondialdehyde, nitric oxide, IL-6, and matrix metalloproteinases-2 and − 9 while increasing superoxide dismutase and IL-10 in the sciatic nerve. Another study found that liraglutide decreased diabetic neuropathy-induced microglial cell activation in the cerebral cortex and thalamus of rats by reducing the expression of the NLR family pyrin domain containing 3 (NLRP3) protein in brain microglia [47]. The analgesic effects of liraglutide were not observed in mice with constitutively active glycogen synthase kinase-3 beta (GSK3β), as activation of GSK3β promotes the activation of the NLRP3 inflammasome [48]. In another model of diabetic neuropathy in rats, significant improvements in pain, oxidative stress, and inflammatory markers of the sciatic nerve were demonstrated following oral administration of amitriptyline and subcutaneous liraglutide, as well as with concurrent oral administration of amitriptyline and liraglutide loaded into proniosomal formulations [49]. PKF275-055, an analogue of vildagliptin and a DPP-4 inhibitor, counteracted changes in Na⁺/K⁺-ATPase activity, nerve conduction velocity, and nociceptive thresholds in diabetic rats [50]. PKF275-055 restored mechanical sensitivity thresholds by approximately 50% and progressively improved thermal responsiveness, suggesting its potential as a therapeutic agent for diabetic neuropathy.

### Visceral pain and irritable bowel syndrome

Seven studies investigated the role of GLP-1 in visceral pain and IBS [51–57]. GLP-1R-like immunoreactivity is present in the innervation of the human colon and is increased in biopsies from individuals with IBS [51]. Administration of GLP-1 and exendin-4 promoted neurite outgrowth in cultured dorsal root ganglion (DRG) neurons, presumably explaining the increased nerve fibers observed in biopsies of IBS individuals [51]. While adenosine triphosphate (ATP) signaling was enhanced, capsaicin sensitivity was unaffected by the acute application of exendin-4 in cultured DRG neurons, suggesting GLP-1R role in modulating gut motility rather than pain signaling. In a rat model of IBS, intraperitoneal administration of exendin-4 normalized stress-induced defecation and visceral pain sensitivity by modulating enteric neuronal function through GLP-1 receptors in submucosal and myenteric ganglion neurons [52]. These benefits appeared to stem from the modulation of enteric neuronal function and tight junction expression. Exendin-4 administration did not affect anxiety-like behaviors regulated by the central nervous system. Another study examined exendin-4 effect on visceral hypersensitivity in colon-sensitized rats [53]. Rats treated with colonic acetic acid infusions exhibited low plasma GLP-1 levels and high serotonin levels in plasma and colonic tissues. Exendin-4 administration reduced visceral hypersensitivity in a dose-dependent manner and decreased serotonin levels in the colon. Post-treatment, the expression of the serotonin reuptake transporter (SERT) significantly increased, while tryptophan hydroxylase-1 (TPH-1) expression significantly decreased in colonic tissue, suggesting exendin-4 mitigates visceral hypersensitivity by upregulating SERT and downregulating TPH-1. In a model of lipopolysaccharide (LPS)-induced visceral hypersensitivity and repeated water deprivation stress in rats, liraglutide reduced visceral allodynia by inhibiting proinflammatory cytokine production and attenuating intestinal permeability [54]. Specifically, liraglutide blocked increased IL-6 levels in colonic mucosa via a nitric oxide-mediated response. In rectosigmoid biopsies from patients with constipation-predominant IBS and healthy controls, GLP-1R was significantly downregulated in IBS individuals compared to controls [55]. Serum GLP-1 levels were significantly lower in IBS individuals and negatively correlated with abdominal pain scores. ROSE-010, a GLP-1 analog, was tested in a randomized, placebo-controlled clinical trial for treating acute pain in IBS patients [56]. As a neuronal GLP-1R agonist, ROSE-010 increases smooth muscle motility in the gastrointestinal tract. Subcutaneous administration of ROSE-010 (100 µg and 300 µg) was well-tolerated and more effective than placebo [56]. Pain relief was dose-dependent, with the best effect observed at 120 min after a 300-µg injection. Patients with at least four abdominal pain attacks per month, each lasting at least two hours, experienced significant pain relief as early as 20 min post-administration. The most pronounced improvements were in patients with frequent constipation compared to those with frequent diarrhea. More patients were satisfied with ROSE-010 and considered it superior to previous treatments for IBS. Female participants responded more positively than males, while age and BMI did not influence treatment response [57].

## Discussion

Our systematic review of GLP-1 role in headache and pain disorders reveals a promising landscape for its application beyond traditional metabolic uses. The potential of GLP-1R agonists as multi-faceted therapeutic agents extends their benefits to various pain conditions, including inflammatory, neuropathic, visceral pain, and headaches (Table 3). GLP-1R activation can downregulate pro-inflammatory mediators and shift macrophage phenotypes toward anti-inflammatory profiles [15–17]. However, the translation to clinical efficacy in human osteoarthritis remains ambiguous, as weight loss appears to play a substantial role in pain alleviation [21, 22]. While the mechanistic insights are encouraging, further clinical investigations are needed to isolate the direct analgesic effects of GLP-1R agonists from those mediated by weight loss. GLP-1 appears to modulate inflammatory pathways and reduce intracranial pressure through direct actions on the choroid plexus in the context of migraine and IIH [23–25]. A clinical trial and a case-control study highlighted the effectiveness of GLP-1R agonists in reducing headache frequency and intracranial pressure in individuals with IIH, independent of weight loss [26, 27]. These findings suggest a novel therapeutic pathway for IIH management, where GLP-1R agonists may offer dual benefits in weight reduction and headache relief [58]. An international multicenter trial is currently underway to validate the effects of GLP-1R activation in active IIH (NCT05347147). The role of GLP-1R in neuropathic pain, particularly diabetic neuropathy, is supported by preclinical evidence. GLP-1R agonists exert their effects by modulating microglial activity and inflammatory cytokine production in the spinal cord. Following peripheral nerve injury, GLP-1R expression is upregulated in microglial cells of the spinal dorsal horn [31]. The activation of GLP-1R promotes the release of β-endorphins, which mediate analgesic effects through µ receptors at the neuronal level [39, 42]. The reduction of inflammatory signaling may play an additional role in the analgesic effects of GLP-1R agonists against neuropathic pain [32, 34, 35]. Notably, these effects are more effective for chronic rather than acute pain and are achieved without the development of tolerance [31, 36, 38, 40–42]. Furthermore, the ability of GLP-1R agonists to ameliorate neuropathy-related complications, such as cognitive deficits, underscores their broader neuroprotective benefits [43, 44]. GLP-1R agonists impact visceral pain, with specific relevance to IBS. The modulation of enteric neuronal function and serotonin signaling by GLP-1R agonists contributes to reduced visceral hypersensitivity and pain [52, 53]. The clinical trial with ROSE-010 demonstrated its efficacy in acute pain relief for IBS patients and correlation between serum GLP-1 levels and abdominal pain severity further supports the role of GLP-1R agonists in managing visceral pain. Despite promising research, it is important to exercise caution when considering the potential benefits of GLP-1 agonists beyond diabetes and obesity. The current trend of uncontrolled use of these medications may obscure potential harms to patients. Our understanding of the long-term safety of GLP-1 agonists remains limited, a concern that is particularly pertinent for chronic conditions such as pain and headaches. Some populations, such as adolescents with migraine who suffer from comorbid obesity, may be of interest for further study, as GLP-1 agonists could potentially relieve pain while also addressing obesity [59]. Properly designed randomized controlled trials are necessary to elucidate these aspects and ensure that the use of GLP-1 agonists in these contexts is both safe and beneficial.

**Table 3:** Human studies evaluating the analgesic effects of glucagon-like peptide 1 receptor (GLP-1R) agonists in headache and pain disorders

Study	Year	Study design	Study drug	Study duration	Disease	Study participants	Main findings
Gudbergsen et al. [21]	2021	Randomized controlled trial	Liraglutide 3 mg/daily	52 weeks	Knee osteoarthritis	156	Liraglutide did not reduce knee pain compared to placebo
Zhu et al. [22]	2023	Observational multicentre study	GLP-1R agonists	At least 5 years	Knee osteoarthritis	> 40 000	Cartilage loss velocity and incidence of knee surgery was lower in individuals treated with GLP-1R agonists
Krajnc et al. [26]	2023	Case-control study	Semaglutide and liraglutide	6 months	Idiopathic intracranial hypertension	39	Reduction in monthly headache days and acetazolamide dosage were higher in individuals treated with GLP-1R agonists
Mitchell et al. [27]	2023	Randomized controlled trial	Exenatide 10 µg twice daily	12 weeks	Idiopathic intracranial hypertension	15	Exenatide lowered intracranial pressure compared to placebo
Hellström et al. [55]	2009	Randomized controlled trial	100 and 300 µg ROSE-010	2 years and 9 months	Irritable bowel syndrome	99	Twice as many patients were responders in the primary efficacy endpoint after both ROSE-010 injections compared to placebo
Touny et al. [56]	2022	Substudy of earlier data	100 and 300 µg ROSE-010	2 years and 9 months	Irritable bowel syndrome	166	Female participants are more likely than males to respond to ROSE-010 to achieve meaningful pain relief

### Limitations

In our systematic review, we did not prioritize the risk of bias evaluation. Our focus was on mapping existing research, identifying trends, and discussing potential directions for further studies. This approach aligns with the goals of a systematic review into a novel research area, where the primary aim is to gather and describe existing knowledge rather than to rigorously assess study quality or synthesize outcomes quantitatively. Another limitation is the broad search strategy employed, using the terms “GLP-1” and “pain”. We did not incorporate more specific keywords related to distinct pain disorders, such as “headache” or “diabetic neuropathy”. While this approach ensured a comprehensive inclusion of relevant articles and provided a general overview of the role of GLP-1 in pain, it may have limited the depth of evidence on specific pain conditions. Future studies should focus on targeted searches to obtain more refined evidence on the relationship between GLP-1 and individual pain disorders.

### Future directions

Future research directions for the use of GLP-1R agonists in the treatment of pain and headache disorders should focus on conducting human trials to evaluate their efficacy and safety, particularly in individuals with comorbid obesity [60]. In addition, detailed mechanistic studies are necessary to elucidate the specific pathways through which GLP-1R agonists exert their analgesic effects, independent of weight loss and metabolic improvements. Exploring the synergistic potential of GLP-1R agonists with other analgesic agents could enhance therapeutic outcomes and reduce the necessity for high-dose monotherapy. Investigating differential responses based on patient demographics, genetic profiles, and specific pain conditions will also be crucial in developing personalized treatment strategies, thereby optimizing the therapeutic benefits of GLP-1R agonists for diverse patient populations. The engineering of bimodal molecules that integrate GLP-1R agonism with other pain-related mechanisms, such as N-methyl-D-aspartate (NMDA) receptor antagonism, may be especially beneficial for pain management [61].

### Conclusions

GLP-1R analogs represent a promising frontier in the treatment of various pain disorders. Their diverse biological effects, coupled with a growing body of preclinical and clinical evidence, support the potential for GLP-1R agonists to be integrated into multidisciplinary pain management protocols. Continued research and clinical trials will be pivotal in fully realizing their therapeutic potential and establishing their role in pharmacological practice.

## Data Availability

No datasets were generated or analysed during the current study.

## Abbreviations

AD: Alzheimer’s disease
AMPK: adenosine monophosphate-activated protein kinase
ATP: adenosine triphosphate
BMI: body mass index
cAMP: cyclic adenosine monophosphate
DRG: dorsal root ganglion
DPP-4: dipeptidyl peptidase 4
GFAP: glial fibrillary acidic protein
GLP-1: glucagon-like peptide 1
GLP-1R: glucagon-like peptide 1 receptor
GSK3β: glycogen synthase kinase-3 beta
Iba-1: ionized calcium-binding adapter molecule 1
IBS: irritable bowel syndrome
IIH: idiopathic intracranial hypertension
IL-1β: interleukin 1β
IL-4: interleukin 4
IL-6: interleukin 6
IL-10: interleukin 10
LPS: lipopolysaccharide
NF-κB: nuclear factor-κB
NLRP3: NLR family pyrin domain containing 3
NMDA: N-methyl-D-aspartate
PD: Parkinson’s disease
PI3K/Akt: phosphoinositide 3kinase/serine-threonine kinase
PKA: protein kinase A
SERT: serotonin reuptake transporter
SM: shanzhiside methyl ester
TNF-α: tumor necrosis factor alpha
TEN: teneligliptin
TPH-1: tryptophan hydroxylase-1
